# Stabilization of Kidney Graft Function Following SGLT2 Inhibitor Treatment in Non-Diabetic Kidney Transplant Recipients

**DOI:** 10.3389/ti.2025.15872

**Published:** 2026-01-12

**Authors:** Diana Rodríguez-Espinosa, Ricardo Parra, Blerina Mataj, Jonay García, Elena Cuadrado-Payán, Vicens Torregrosa, Nuria Esforzado, Ignacio Revuelta, Pedro Ventura-Aguiar, David Cucchiari, Enrique Montagud-Marrahí, Alicia Molina-Andújar, Carolt Arana, Ángela González, José Jesús Broseta, Fritz Diekmann

**Affiliations:** Department of Nephrology and Renal Transplantation, Hospital Clínic of Barcelona, Barcelona, Spain

**Keywords:** chronic allograft dysfunction, chronic kidney disease (CKD), kidney allograft, nephroprotection, SGLT2 inhibitors

Dear Editors,

Sodium-glucose cotransporter 2 inhibitors (SGLT2i) have demonstrated nephroprotective effects in patients with chronic kidney disease (CKD) regardless of diabetes. However, their efficacy and safety in non-diabetic kidney transplant recipients (KTR) is limited [1, 2].

This was a single-center, retrospective study of adult KTR patients prescribed an SGLT2i between January 2021 (when the drug was first approved for its use in our health area) and September 2024, followed through September 2025, to analyze the annual decline rate in estimated glomerular filtration rate (eGFR) before and after SGLT2i initiation. Only patients with a transplant vintage of at least 12 months before SGLT2i initiation were included. Those who discontinued treatment or experienced an episode of rejection or recurrent glomerulonephritis within the preceding 12 months were excluded to focus on clinically stable recipients not recovering from acute events or under the influence of prophylactic trimethoprim–sulfamethoxazole, which can transiently affect serum creatinine levels. This study was conducted in accordance with the principles of the Declaration of Istanbul on Organ Trafficking and Transplant Tourism and the ethical guidelines of the host institution.

The primary endpoint was the within-patient difference in the 12-month eGFR slope [3] before and after receiving an SGLT2i, expressed in mL/min/1.73 m^2^ and calculated with the CKD-EPI formula [4]. The secondary endpoint was the change in the urinary albumin-to-creatinine ratio (UACR), if available over the same period and ≥30 mg/g at baseline, expressed as mg/g. eGFR slopes were calculated for each patient and compared using the Wilcoxon signed-rank test. A linear mixed model for repeated measures was used to analyze changes in eGFR over time. A general linear model was used to adjust the post-SGLT2i eGFR slope for covariates.

Regarding UACR reduction, the association between time (pre-vs. post-SGLT2i) and the probability of response was evaluated using a generalized estimating equation model with a binomial distribution, accounting for repeated measures within subjects. Odds ratios (OR) with 95% confidence intervals (CI) were estimated. A two-tailed *p*-value < 0.05 was considered statistically significant. Analyses were performed using SPSS Statistics (version 25.0, IBM Corp, Armonk, NY, United States).

A total of 82 non-diabetic KTR were prescribed an SGLT2i during the mentioned period. Fifteen patients were excluded due to treatment discontinuation, two had a biopsy proven graft rejection and three a glomerular disease recurrence within 12 months of treatment initiation, and four lacked sufficient paired data. The reasons for discontinuation included: death (2), graft loss (5), renal function decline (1), diarrhea (1), erythrocytosis (1), dyspnea (1), intolerance (2), non-adherence (1), and other/unknown reasons (1). Baseline demographic and clinical characteristics of the 58 included patients are detailed in Table 1.

**TABLE 1:** Baseline and clinical characteristics of the study population.

Variable	Study population
	n = 58
Recipient age, median (IQR)	50.5 (39.3–61)
Donor age, median (IQR)	53 (43–59)
Recipient male sex, n (%)	34 (58.6)
Recipient BMI, Kg/m^2^, median (IQR)	26.1 (21.6–29.9)
Donor BMI, Kg/m^2^, median (IQR)	25.8 (23.5–28.6)
Immunosuppression, n (%)	
MPA, n (%)	36 (62.1)
mTORi, n (%)	21 (36.2)
CNI, n (%)	48 (82.8)
Belatacept, n (%)	6 (10.3)
RAASi, n (%)	33 (56.9)
ACEi, n (%)	9 (15.5)
ARB, n (%)	24 (41.4)
Transplant number	
First, n (%)	45 (90.4)
Second, n (%)	7 (9.8)
Third, n (%)	4 (5.9)
Fourth, n (%)	2 (3.9)
Type of donor	
Living donor, n (%)	25 (43.1)
Deceased, n (%)	33 (56.9)
DBD, n (%)	24 (41.4)
DCD, n (%)	9 (13.8)
Baseline eGFR, mL/min/1.73m^2^, median (IQR)	43 (34–52)
Baseline UACR, mg/g, median (IQR)	145 (10–567)
GN recurrence	0
BPAR	17 (29.3)
TCMR, n (%)	12 (70.5)
AMR, n (%)	2 (11.8)
caAMR, n (%)	3 (17.6)

AMR, antibody-mediated rejection; BMI, body mass index; BPAR, biopsy-proven allograft rejection; CI: confidence interval; caAMR, chronic-active AMR; CNI, calcineurin inhibitor; DBD, dead brain donor; DCD, donor after circulatory death; eGFR, estimated glomerular filtration rate; IQR, interquartile range; MPA, mycophenolic acid; mTORi, mammalian target of rapamycin inhibitor; RAASi, renin-angiotensin-aldosterone inhibitors; TCMR, T-cell mediated rejection. Patients with biopsy-proven rejection or glomerulonephritis recurrence within the 12 months prior to SGLT2i initiation were excluded from the study.

In the repeated-measures analysis of eGFR, there was a significant overall effect of time (F = 6.3, p = 0.003). Pairwise comparisons adjusted for immunosuppressive treatment and the use of renin-angiotensin-aldosterone inhibitors (RAASi) showed a progressive decline in kidney function during the pre-SGLT2i period (p = 0.002), while there was no statistical difference between baseline eGFR and 12 months after starting the medication (p = 1). The median annual eGFR slope in the pre-SGLT2i period was −3.5 (−7 to 0) mL/min/1.73 m^2^, while in the post-SGLT2i period it was −1 (−5.5 to 4) mL/min/1.73 m^2^ (p = 0.003). In the adjusted general linear model, there was no correlation between slope and RAASi, age, transplant vintage, rejection, or immunosuppressive regimen.

In the repeated-measures analysis of the 30 patients with baseline UACR ≥30 mg/g, there was no significant overall difference between periods (F = 2.4, p = 0.067). However, the odds of achieving a ≥30% reduction in UACR increased after initiation of an SGLT2i. In the 12 months preceding treatment, 14% of patients (3/21) achieved a ≥30% UACR reduction, compared with 47% (14/30) at 12 months post-initiation, corresponding to an OR of 5.3 (95% CI 1.2–23.3; p = 0.027).

Regarding adverse events, four patients developed genitourinary fungal infections, with two events occurring before and two after SGLT2i initiation. In addition, five patients experienced a UTI, four before and one after the start of SGLT2i therapy.

Our findings contribute to the expanding, albeit still limited, body of evidence regarding the use of SGLT2i in KTR. Most previous studies have concentrated on diabetic KTR [5, 6] In contrast, pivotal trials involving non-transplant CKD populations [1, 2] have shown that SGLT2i consistently slow the decline in kidney function across both diabetic and non-diabetic patients.

Our cohort demonstrates that kidney function stabilized after initiation of SGLT2i, suggesting that SGLT2i potential nephroprotective effects may extend beyond diabetes. It is important to note that there is currently no universally accepted benchmark for eGFR slope in KTR, with published estimates varying widely from less than 1 to over 7 mL/min/1.73 m^2^ per year, depending on the timing and selection of patients [7, 8]. Thus, the observed results should be interpreted with caution. However, the difference between pre- and post-treatment annual eGFR slopes exceeded 0.75 mL/min/1.73 m^2^, a magnitude regarded as clinically meaningful in terms of lowering the risk of CKD progression [3].

Proteinuria reduction represents a key mechanism behind the nephroprotective effects of SGLT2i. Lim et al. reported greater reductions in proteinuria among KTR who experienced an early decline in kidney function after starting SGLT2i [6]. In contrast, we observed no significant overall change in UACR; however, their cohort consisted of diabetic patients, who may have had higher baseline proteinuria than ours (they did not report baseline UPCR values). Notably, patients in our study were more likely to achieve a ≥30% reduction from baseline UACR, which represents the surrogate endpoint recommended by both the American Diabetes Association and the KDIGO guidelines [9, 10].

This study has limitations. It is a retrospective, single-center analysis with a small sample size, which limits generalizability. The exclusion of patients with recent rejection or early discontinuation may have introduced selection bias, though it allowed evaluation of a clinically stable cohort. The absence of a control group and heterogeneous transplant vintage also restrict causal inference, and treatment indication was not standardized, introducing potential confounding despite the within-patient design mitigating interindividual variability. Data on the early eGFR dip were unavailable due to follow-up intervals, and UACR data were incomplete, with heterogeneous assessments. The 12-month follow-up captures only short-term outcomes, and tacrolimus levels were not collected, although prior studies suggest no relevant pharmacologic interaction.

No safety concerns were observed, and the absence of discontinuations due to UTI is reassuring.

In summary, SGLT2i may attenuate eGFR decline in non-diabetic KTR, warranting confirmation in larger prospective studies.

## Data Availability

The data analyzed in this study is subject to the following licenses/restrictions: Data can be provided upon reasonable request to the first author. Requests to access these datasets should be directed to DR-E, dmrodriguez@clinic.cat.
